# Cytogenomic description of a Mexican cohort with differences in sex development

**DOI:** 10.1186/s13039-024-00685-1

**Published:** 2024-07-15

**Authors:** Grecia C. Olivera-Bernal, Marlon De Ita-Ley, Edgar F. Ricárdez-Marcial, Luz María Garduño-Zarazúa, Ángel Ricardo González-Cuevas, Omar A. Sepúlveda-Robles, Juan Carlos Huicochea-Montiel, Alan Cárdenas-Conejo, Laura Santana-Díaz, Haydeé Rosas-Vargas

**Affiliations:** 1 Medical Research Unit in Human Genetics, Instituto Mexicano del Seguro Social (IMSS)/Hospital de Pediatría, Centro Médico Nacional SXXI, Ave. Cuauhtémoc 330, 06720 Mexico City, Mexico; 2 Department of Genetics, Instituto Mexicano del Seguro Social (IMSS)/Hospital General Centro Médico Nacional “La Raza”, Mexico City, Mexico; 3 Department of Genetics and Molecular Biology, Centro de Investigación y de Estudios Avanzados, Instituto Politécnico Nacional, Mexico City, Mexico; 4 Department of Paediatric Genetics, Instituto Mexicano del Seguro Social (IMSS)/Hospital de Pediatría, Centro Médico Nacional S XXI, Mexico City, Mexico

**Keywords:** Cytogenetic analysis, Differences in sex development, Chromosomal sex, Cytogenomic, Disorders of sex development

## Abstract

**Background:**

Differences in Sex Development (DSD) is a heterogeneous group of congenital alterations that affect inner and/or outer primary sex characters. Although these conditions do not represent a mortality risk, they can have a severe psycho-emotional impact if not appropriately managed. The genetic changes that can give rise to DSD are diverse, from chromosomal alterations to single base variants involved in the sexual development network. Epidemiological studies about DSD indicate a global frequency of 1:4500–5500, which can increase to 1:200–300, including isolated anatomical defects. To our knowledge, this study is the first to describe epidemiological and genetic features of DSD in a cohort of Mexican patients of a third-level care hospital.

**Methods:**

Descriptive and retrospective cross-sectional study that analyzed DSD patients from 2015 to 2021 attended a Paediatric Hospital from Mexico City.

**Results:**

One hundred one patients diagnosed with DSD were registered and grouped into different entities according to the Chicago consensus statement and the diagnosis defined by the multidisciplinary group. Of the total, 54% of them belong to the chromosomal DSD classification, 16% belongs to 46, XX and 30% of them belongs to the 46, XY classification.

**Conclusion:**

The frequency for chromosomal DSDs was consistent with the literature; however, we found that DSD 46, XY is more frequent in our cohort, which may be due to the age of the patients captured, the characteristics of our study population, or other causes that depend on the sample size.

**Supplementary Information:**

The online version contains supplementary material available at 10.1186/s13039-024-00685-1.

## Introduction

Disorders of sexual differentiation or differences in sex development (DSD) are a heterogeneous group of congenital alterations that affect inner and/or outer primary sex characters. These anomalies are characterized by discrepancies between chromosomal, gonadal, and phenotypic sexual determination, which could result from chromosomal, genetic defects, or teratogenic challenges during prenatal development [1–3].

Although most of these conditions do not increase patient mortality, a deficient follow-up leads to psycho-emotional distress that negatively impacts the quality of life of affected subjects [4, 5]. However, there are conditions that can endanger the lives of patients, such as congenital adrenal hyperplasia, in which making an early diagnosis is a priority [5].

Proper management includes determining the DSD etiology; this information is essential to predict the patient's phenotype, the risk of gonadal tumors, and, in some cases, the risk of recurrence in the family [6]. The genetic causes of DSD are broad; they can arise from whole chromosomal alterations to single nucleotide variants or digenic and oligogenic mutations [6–8]. During sex development, A comprehensive list of genes known to be involved in human 46,XY and 46,XX DSD is reported along with their chromosomal locations: it amounts to 62 genes in 46,XY and 61 in 46,XX [7]. However, other modifications in non-coding regulatory sequences, gene-protein interactions, and epigenetic variations can also modify gene expression and increase the risk for these congenital malformations [7, 9].

Although genetic heterogeneity could represent a challenge to obtain a certain diagnosis, the diagnostic algorithm requires the classification of DSD type as an initial step. The Chicago DSD Consensus is a classification in which an attempt has been made to group these entities to standardize a universal definition and diagnosis. This consensus classifies DSD mainly by chromosomal complement but also emphasizes the use of information about differences between sex chromosome alterations, the gonads histopathology, the presence of abnormal paramesonephric ducts or mesonephric embryonic structures derivatives, as well as the metabolic and placental alterations that can modify sexual development [10, 11]. Based on these features, three groups of patients have been described: (1) patients with alterations in sex chromosomes, which include Turner and Klinefelter syndromes; (2) patients with karyotype 46, XX, including congenital adrenal hyperplasia (CAH); and (3) patients with karyotype 46, XY that include androgen insensitivity syndrome (AIS), and 5 alpha reductase deficiency, among others [10].

Although Chicago's classification allows the stratification of patients and their clinical management, it is essential to note that this does not necessarily reflect its etiology, especially in cases with normal karyotypes and discordant sexual phenotypes. DSD diagnosis requires a multidisciplinary framework evaluation due to the difficulty and complexity of establishing a certain diagnosis and determining the approach and treatment, if any [12, 13]. When the 46,XX, or 46,XY karyotype is observed, the use of genomic studies can contribute to identify the origin of the DSD. Unfortunately, even using these strategies, in half of patients, there is no identifiable cause [5, 10].

As these limitations are difficult to surpass, the epidemiological information on these defects is limited. Studies indicate a global DSD frequency of 1:4500–5500 using the atypical genitalia criteria, but this can increase to 1:200–300 when the description of cases includes congenital anomalies of the genitals, such as cryptorchidism or hypospadias [6]. The estimated incidence of chromosomal DSDs is 1:2,500 live infant girls for patients with Turner syndrome and 1:500 live boy births for patients with Klinefelter syndrome [2, 14]. Regarding patients with DSD and karyotype 46, XY, the overall incidence is 1:20,000 births; for patients with ovotesticular DSD, a frequency of 1:100,000 has been estimated [15, 16]. Finally, in patients with DSD 46, XX, it has been described that congenital adrenal hyperplasia (CAH) is the major cause of alterations in female genitalia, showing an overall incidence of 1:14,000–15,000 [17].

Furthermore, the epidemiology is underestimated because only a small proportion of DSDs are officially registered with follow-up at the third level of care [18]. All these limitations represent a challenge to geneticists in DSD counseling, reflecting the importance of continuing the study of patients using genetic, genomic, and epigenetic approaches, but epidemiological assets are also required to improve patient care. This information is vital to developing early diagnosis approaches, adequate treatment, and follow-up, thus making other medical practitioners aware of these congenital entities and their ethical and legal aspects [3, 19, 20].

In international reports, some cohorts of patients with DSD are reported, but in most cases, there are different ways to address them and to identify the etiology [21, 22]. Furthermore, few studies have been carried out in developing countries; in the Mexican population, the studies emphasize a few entities such as Turner, ovotesticular DSD, and AIS, but none of the reports integrates all the DSD diagnoses in a single study [23–26]. Therefore, epidemiological information on these entities is required in populations considering all the DSD patients. The present study describes epidemiological and genetic features of Differences in Sex Development of a cohort of Mexican patients of a third-level care hospital.

## Materials and methods

This is a descriptive and retrospective cross-sectional study of DSD patients recruited from 2015 to 2021, attended in the Paediatric Hospital from Centro Médico Nacional Siglo XXI, IMSS in Mexico City. The diagnosis was made by consensus of a multidisciplinary group formed by medical specialists from the Departments of Endocrinology, Urology, Paediatric Psychiatry, Pathological Anatomy, and Genetics. Due to the epidemiological approach of this study, informed consent was not required, and the ethics committee approved the protocol (R-2019-3603-076). Patients' clinical information was obtained from medical records, and classification was based on the Chicago consensus (2016). Only patients with complete records, including gonads, mesonephric or paramesonephric derivatives evaluation by imaging studies, complete blood count (CBC), blood chemistry tests, serum hormonal profile, and the presence of conventional and/or molecular cytogenetic studies that supported the clinical diagnosis were included in the study.

## Results

One hundred and one patients diagnosed with DSD were registered and grouped into different entities according to the Chicago consensus and the diagnosis defined by the multidisciplinary group (Table 1). Of the total, 71% (n = 71) had an accurate diagnosis, confirmed with biochemical and cytogenetic studies. In the remaining 30% (n = 30), only a clinical diagnosis was detected, and it was not possible to make them a definitive diagnosis; therefore, the karyotype's clinical features and the result determined its classification, see Table 1.

**Table 1:** Distribution of cases analyzed according to the modified Chicago consensus

DSD group N (%)	Subgroup	Karyotype/Clinical type	N
Chromosomal Disorder 55 (54%)	Turner Syndrome	Detailed description in Table 2	34
	Mixed gonadal dysgenesis	Detailed description in Table 3	8
	Klinefelter and variants	47,XXY	2
		49,XXXXY	2
		mos 49,XXXXY/48,XXXX	1
		mos 47,XXY/46,XY	1
		48,XXXY	2
	Chromosomal X polysomy	47,XXX	2
		48,XXXX	1
	Chromosomal Y polysomy	mos 47,XYY/46,XY	2
46,XX 16 (16%)	Congenital Adrenal Hyperplasia	Classical	11
		Atypical Virilizing	2
		Simple Virilizing	1
	Ovotesticular DSD		2
46,XY 30 (30%)	Insensitivity to the action of androgens	Partial Insensitivity	3
		Complete Insensitivity	1
	5 alpha reductase deficiency		2
	Isolated Cryptorchidism		2
	Isolated medial Hypospadias		2
	Congenital Adrenal Hyperplasia	Classical	2
	Persistent Müllerian(s)		1
		Syndromic DSD	5
		Unclassified cases due to difficult clinical diagnosis (in majority PAIS vs Def 5-alpha reductase)	12

DSD: Differences in Sex Development, PAIS: Partial Androgen Insensitivity Syndrome

These patients are complicated in the approach and in making a certain diagnosis, because various symptoms and clinical data are very similar, so the genetic test is highly recommended. However, in our population this test was not possible for economic resources reasons.

DSD with chromosomal/aneuploidy basis of the diagnosed cases represented 54%, whereas patients with the composition of karyotype 46, XY represented 30%, and those with 46, XX, 16% (Table 1). Most DSD patients with chromosomal/aneuploidy had Turner Syndrome, followed by patients with mixed gonadal dysgenesis or sex chromosome polysomy and finally, patients with Klinefelter syndrome, as seen in Table 1. On patients with karyotype 45,X, the majority of them have hypoplastic ovaries with Müllerian(s). In 5 Turner syndrome patients, gonadal streaks with Müllerian(s) were observed, and in two cases, a unilateral ovary with Müllerian(s) was observed. Finally, all the cases with chromosomal mosaics in Turner syndrome presented hypoplastic ovaries with Müllerian(s) (Table 2).

**Table 2:** Characteristics of cases with Turner syndrome

Age at Diagnosis	Karyotype	Gonads
1 m	45,X[30]	M
3 m	45,X[30]	M
8 m	45,X[25]	GSM
10 m	45,X[25]	GSM
11 m	45,X[30]	OM
18 m	45,X[25]	GSM
5.8 yr	45,X[30]	HOM
5.9 yr	45,X[30]	HOM
6.1 yr	45,X[25]	HOM
8.7 yr	45,X[30]	HOM
9 yr	45,X[30]	HOM
9.6 yr	45,X[30]	HOM
14 yr	45,X[25]	HOM
14 yr	45,X[26]	HOM
15 yr	45,X[27]	HSOM
15.3 yr	45,X[30]	HOM
16 yr	45,X[30]	HOM
14.6 yr	45,X,inv(9)(p13q13)[30]	GSM
12.1 yr	mos 45,X[13]/46,XX[37]	HRO, AOLM
10.2 yr	mos 45,X[1]/46,XX[44].nuc ish(DXZ1 × 1,DYZ3 × 0)[5] /(DXZ1 × 2,DYZ3 × 0)[495]	OM
15 yr	mos 45,X[10]/46,XX[20]	HOM
5.9 yr	mos 45,X[4]/46,XX[30]	HOM
14 yr	mos 46,X, + mar[33]/45,X[17].nuc ish(DXZ1 × 1,DYZ3 × 0)[486]/(DXZ1 × 2,DYZ3 × 0)[14]	GSM
14 yr	mos 45,X[27]/46,X,inv(X)(q28q13)[13]	HOM
9 yr	mos 45,X[3]/46,XX[72]	HOM
10.2 yr	mos 46,X,r(X)[30]/45,X[6]	HOM
6.7 yr	mos 45,X[17]/46,X,r(X)[16]	HOM
9 yr	mos 46,X,r(X)(p11q22)[28]/45,X[15]	HOM
8 yr	mos 46,X,i(X)(q10)[17]/45,X[13]	HOM
12.7 yr	46,X,i(X)(q10)[25]	GSM
9.9 yr	46,X,del(X)(p22.3)[30]	HOM
8 yr	mos 46,X,i(X)(q10)[17]/45,X[13]	HOM
16.9 yr	mos 45,X[20]/47,X,i(X)(q10)[12]/46,XX[8]	SOWM
8 yr	mos 45,X[44]/46,X,+idic(Y)(p11.2)[6].ish idic(Y)(DYZ1++)[4]. nuc ish(DXZ1 × 1,DYZ1 × 0)[435]/(DXZ1 × 1,DYZ1 × 2)[29] /(DXZ1 × 1,DYZ1 × 1)[59] /(DXZ1 × 2,DYZ1 × 0)[2]	HOM

HOM: Hypoplastic ovaries with Müllerian(s), GSM: Gonadal Streaks with Müllerian(s)

HSOM: Hypoplastic single ovary with Müllerian(s); OM: Ovaries with Müllerian(s);

M: Müllerian(s); HRO: Hypoplastic right ovary; AOLM: Absence of left ovary with Müllerian(s);

SOWM: Single ovary without Müllerian(s)

In the case of patients with Klinefelter syndrome, all the subjects presented small testicles with mesonephric/Wolffian(s), regardless of the different cytogenetic variants diagnosed. Karyotypes 47, XXY; 48, XXXY; 49, XXXXY, and mosaics were observed in these patients (Table 1). Finally, in cases diagnosed with mixed gonadal dysgenesis, a gonadal-wide variability presentation was found in each patient without directly correlating with the percentage of mosaic in the blood cell line (Table 3).

**Table 3:** Characteristics of cases with mixed gonadal dysgenesis

Age at diagnosis (years)	Karyotype	Gonads	Genitals	Assigned Sex
1.2 yr	mos 45,X[18] /46,XY[37]	Unilateral inguinal testis with Wolffian(s)	Atypical: phallus length 1.3 cm, urethral opening at the base, rough labioscrotal folds, pigmented, fused in lower region, with sacral pit, palpable gonad in inguinal left region	Female
7.9 yr	mos 45,X[9]/46,XY[21]	Unilateral inguinal testis with Müllerian(s)	Hypovirilized: perineal hypospadias, penile length 4.5 cm, penial cord, palpable gonad in inguinal left region	Male
5 yr13	mos 45,X[26]/46,XY[24]	Unilateral inguinal testis without Wolffian(s)	Atypical: phallus length 20.5 mm, labioscrotal folds, fused alt the center region, urethral opening at ventral region, proximal hypospadias, left gonad in inguinal region, no right gonad visible	Male
6.5 yr	mos 45,X[13] /46,XY[32]	Testis with Müllerian(s)	Hypovirilized: penoscrotal hypospadias, penile length 3.1 cm, palpable gonad in left inguinal region	Male
13 yr	mos 45,X[25]/46,XY[26]	Testis with Wolffian(s)	Atypical: penoscrotal hypospadias, urethral diverticulum, phallus length palpable gonad in left inguinal region	Male
4.4 yr	mos 45,X[15]/46,XY[30]	Right testis and left ovotestis with Müllerian(s)	Atypical: scrotalized labia majora, urethral opening under the clitoris, gonads in abdominal cavity	Female
1.7 yr	mos 45,X[2]/46,XY[98]	Unilateral testis with cystic degeneration	Atypical: penoscrotal hypospadias, phallus length 3.5 cm, shawl scrotum, rough and hyperpigmented, left gonad in inguinal region, no palpable gonad in right side	Male
5 m	mos 45,X[39]/46,XY[11]	Gonadal streaks with Müllerian(s)	Atypical: labioscrotal folds, hyperpigmented, phallus length 3.7 cm, penoscrotal hypospadias, visible introitus, gonadal streaks in abdominal cavity	Male

In DSD 46, XX patients, 14/16 (87.56%) correspond to the clinical diagnosis of congenital adrenal hyperplasia (CAH). Of these, 11/14 were classical variants, 2/14 were virilizing atypical variants, and one case (7.14%) was described as a simple virilizing variant by clinical data and biochemical profile. CAH observed a degree of genital virilization (Prader II-IV) in all patients. In 4/14 patients, female genitalia with clitoromegaly were described, while in 10/14, atypical genitalia were observed. The diagnosis was established mainly based on clinical and laboratory information, so these patients did not have genetic test confirmation at the time (see Table 4).

**Table 4:** Patients with Congenital Adrenal Hyperplasia

Patient karyotype	Age	17-OHP and cortisol serum levels	Clinical features
46,XX	3 mo	67 ng/ml, cortisol 25 nmol/L	Atypical genitalia, scrotalized folds, hyperpigmented, fused labia with ovaries and uterus. Prader 2
46,XX	2.3 yrs	45 ng/ml, cortisol 52.5 nmol/L	Atypical genitalia, scrotalized folds, hyperpigmented, urogenital sinus, with ovaries and uterus Prader 3
46,XX	29 yrs	10 ng/ml, cortisol 101.2 nmol/L Probably Atypical virilized	Clitoromegaly at 3 yrs. Uterus and ovaries with a cyst in left ovary. Prader 2
46,XX	4 mo	1273 ng/ml, cortisol 32.3 nmol/L	Atypical genitalia with Hyperpigmentation in armpits and genitalia, fused labia, phallus 4 cm with uterus and ovaries. Prader 4
46,XX	1 yr	85 ng/ml, cortisol 44.6 nmol/L	Atypical genitalia with scrotalized and fused folds. Uterus and ovaries present. Prader 3
46,XX	9 mo	110 ng/ml, cortisol 99.5 nmol/L	Atypical genitalia with Scrotalized and hyperpigmented folds. Uterus and ovaries. Prader 2
46,XX	9 mo	476 ng/ml, cortisol 20.1 nmol/L	Atypical genitalia, phallus 1.5-2 cm, urinary meatus at base, labia majora fused, no palpable gonads. Uterus in ultrasound, no identifiable gonads. Prader 3
46,XX	3.8 yrs	270 ng/ml, cortisol 50.2 nmol/L	Clitoromegaly, dehydration, no palpable gonads. Uterus and ovaries in ultrasound. Prader 2
46,XX	5 mo	49.5 ng/ml, cortisol 69.5 nmol/L	Atypical genitalia, hypoplasia of labia minora, patent introitus and no palpable gonads. Uterus and ovaries in pelvic cavity. Prader 2
46,XX	3.1 yrs	22.5 ng/ml, cortisol 165 nmol/L Probably Simple virilizing	Clitoris 2.5 cm × 4.5 cm, no palpable gonads, no hyperpigmentation. Uterus and ovaries present. Prader 2
46,XX	1.2 yrs	1468 ng/ml, cortisol 32.1 nmol/L	Atypical genitalia, hyperpigmentation, phallus 3.5 cm × 4.8 cm, no palpable gonads. Uterus and ovaries present. Prader 4
46,XX	5.6 yrs	8.4 ng/ml, cortisol 180.3 nmol/L Probably Atypical virilized	Progressive clitoromegaly Prader 2, hyperpigmented armpits and hypertrichosis
46,XX	1.3 yrs	52.3 ng/ml, cortisol 76.7 nmol/L	Atypical genitalia with fused labia Prader 3. Uterus and ovaries
46,XX	2 mo	58.7 ng/ml, cortisol 50.4 nmol/L	Atypical genitalia with hyperpigmented folds, phallus 3.3 cm × 4.2 cm. Prader 3
46,XY	1 mo	1235.1 ng/ml, cortisol 20.2 nmol/L	Adrenal crisis, normal external male genitalia
46,XY	2 mo	1115.8 ng/ml, cortisol 19.3 nmol/L	Dehydration crisis with normal external male genitalia

Furthermore, the last 2/16 were also observed with ovotesticular DSD, bilateral ovotestis, and Müllerian(s) were also observed. One of them was assigned as male with close follow-up by child psychiatry to monitor his behavior and reduce the probability of gender dysphoria in the future.

On the other hand, the DSD 46,XY represented a complex analysis since only in some of them was it possible to confirm the clinically proposed diagnosis molecularly. For patients with AIS and 5 alpha reductase deficiency, the diagnosis was made using only clinical features, through quantifications of the biochemical profile and imaging.

For this group, it is not possible to determine the frequency of the underlying clinical entities using only the studies we have access to so far, since many cases do not have a precise genetic diagnosis and require a complementary molecular genetic approach.

Among the cases analyzed, twelve cases stand out because they present a normal karyotype and genital ambiguity, and, as part of the study of the mechanism involved and diagnosis, the result of the cytogenomic study by microarrays was available. Each of them is briefly described below in supplementary Table 5. Of these cases, all had karyotypes without visible numerical or structural alterations at this level of resolution 450–550 bands, four presented a dysmorphic phenotype and affectations to different organs and systems, so they were classified as syndromic. Of these cases, one patient was diagnosed with 22q11.2 deletion syndrome.

The most frequent sexual assignment found in patients with complex abnormalities was analyzed and determined as female (56%). In five patients, the sex assignment was changed after the multidisciplinary approach, of which four were changed from male to female and one case from female to male, corresponding to one case of DSD 46, XY.

## Discussion

This study describes the type and frequency of chromosomal and clinical alterations in a group of patients diagnosed with DSD in a tertiary care hospital [27, 28]. In Mexico, there are some guidelines for the follow-up of chromosomal DSD, particularly Turner syndrome; however these have not been up to date since the early 2000s [29]. For the rest of the DSD, there is no epidemiological information. To our knowledge, this study is a unique cohort study of patients with DSD in our country due to the integral approach of the patients.

In the international literature, chromosomal DSDs are the most frequent within the general classification of differences in sex development [12, 13]. In our cohort of patients, it coincides that the most frequent alterations belong to the group of chromosomal DSDs, specifically Turner Syndrome. In this sense, it has been observed that 40–50% of patients diagnosed with Turner syndrome have a 45, X karyotype [30]; in our study, it occurred in 53% of patients with this diagnosis, that is consistent with described in the international reports.

On the other hand, it has been described that karyotype 47, XXY is presented in 80–90% of patients with Klinefelter syndrome and chromosomal variants are much less frequent, with an incidence of 1:18,000 to 1:100,000 births. These cases are generally associated with severe alterations such as intellectual disability and behavioral alterations [31, 32]. In our cohort, it occurred in 2 of 8 patients, corresponding to 29% of patients with this diagnosis, while chromosomal variants of this entity occurred in 5 cases (71%). The discrepancy concerning the percentages reported in the literature could be related to the size of the cohort, or the age of the patients in our cohort (ranges 8–17 yrs) since these chromosomal variants may have more severe manifestations, as mentioned, are usually referred early to different levels of care.

It should be noticed that in our cohort, chromosomal DSD are followed in frequency by DSD 46, XY with 30% of patients diagnosed, which is not expected for this type of entity since the international papers reports a lower frequency (from 5000 to 10,000 fewer cases), compared to DSD 46, XX [8, 12, 13, 33]. These results may be due to the selection bias of the subjects because patients from a third-level unit were only referred by second-level care units, and are sometimes diagnosed and treated at the second level, and are no longer referred for care. Another possibility would be that DSD 46, XY are more frequent in our population, which would have a fundamental implication for the approach and treatment of these patients. These cases require closer secondary follow-up due to the high probability of developing germline tumors throughout life (20–30%) [34], mainly in the first years, which is modified depending on the specific diagnosis. To analyze the cause of this discrepancy with the literature, it is necessary to extend the size of the population analyzed.

Among the syndromic cases, it is noteworthy the presence of a patient with 22q11.2 deletion syndrome, with genital and gonadal ambiguity, and an absence of Müllerian(s) and gonads. It has been reported that these alterations are not associated with 22q11.2del. Therefore, the overlap of two different genetic entities could be the etiology of this phenotype and do more research.

It is essential to mention that 30% of analyzed patients do not have a certain diagnosis, although they presented a presumptive clinical diagnosis. However, to establish most of the diagnoses in DSD 46, XY studying the genetic anomalies associated with these conditions is essential because they overlap clinical features and are inherited under different patterns [35, 36]. To reach 50% diagnosis and improve genetic counseling for patients with DSD is necessary to use molecular and sequencing approaches [37]. These molecular studies (exome, Sanger sequencing) are planned to be carried out in the next phase of this project, which will have its own registry in our institute and will receive separate funding.

One limitation of this study, is that the cohort is from a paediatric hospital, which could be part of a restriction to get more clinical data about DSD in our country; therefore, expanding the age range of patients included in future studies is desirable. However, many patients with DSD are not detected until adulthood, when they manifest primary amenorrhea or infertility [6, 30].

Finally, the average age at which the DSD diagnosis was made in our cohort varies depending on the specific entity. In the entities in which atypical genitalia was observed at birth, the followship was initiated before 12 months of age. However, in entities such as Turner Syndrome, which does not display external genitalia abnormalities the average consultation age was 8.2 years, above the international recommendations for initiating an adequate approach [8, 38]. The origin of this deficiency could lie in the lack of specialized knowledge about these entities for primary care physicians; if the whole clinical picture is not identified in the early stages of development, diagnosis and timely management can be delayed, so it is crucial to reinforce the knowledge of DSD in the training of health professionals. Although it is a complex issue, an early diagnostic suspicion will offer support for early management, treatment, and genetic counseling that will benefit the health of the DSD patient and their family and reduce the possibility of associated complications depending on the specific pathology.

## Conclusion

A descriptive study of the DSD analyzed in seven years was carried out in a tertiary institution. The frequency for chromosomal DSDs was consistent with the literature; however, we found that DSD 46, XY is more frequent in our cohort, which may be due to the age of the patients captured, the characteristics of our study population, or other causes that depend on the selection bias of the subjects.

The optimal management of patients with diagnostic DSD requires a multidisciplinary team and molecular studies, promptly achieving the most appropriate and smooth sexual assignment during our population's first years of life. Finally, this study contributes to and substantiates the need to create an epidemiological database about the genetic origin of the different DSDs in our population. Using this database could improve our population's diagnosis and management of patients. Additionally, this information justifies the need to implement approach guidelines in our country for these entities and a national database that allow the advancement of new research in treatments that benefit and improve the quality of life of these individuals.

### Supplementary Information

Additional file 1

## Data Availability

The database can be consulted by permission in the following access: https://docs.google.com/spreadsheets/d/138iiHh3rXXWjestBnQEP8Qeq-5iQmlpcZBYmjtxD-0Q/edit?usp=sharing.
