# Supplementary material for: Cytogenomic description of a Mexican cohort with differences in sex development
Source: Mol Cytogenet. 2024 Jul 15;17:16. doi: 10.1186/s13039-024-00685-1 (PMC11251293; doi:10.1186/s13039-024-00685-1)
Supplement: Supplementary file 1 — Additional file 1 [file 13039_2024_685_MOESM1_ESM.docx]

| Table 5. Cases with atypical genitalia and difficult clinical diagnosis | | | | | | | |
| --- | --- | --- | --- | --- | --- | --- | --- |
| Age | **Karyotype** | **Arrays** | **Syndromic** | **Gonads** | **Genitalia** | **Assigned sex** | **Diagnosis** |
| 6 m | 46,XY[30] | arr[GRCh37] Xq27.2(140673728_140783042)x0 2p11.2(87126323_87309821)x1, 2q11.2(97733013_98118115)x1, 6p25.3(257340_381137)x1, 11p11.12q11(51581311_54758019)x1, 14q11.2(22598597_22944507)x1, 14q32.33(106329184_106715317)x3, 17p11.1q11.1(22227470_25274363)x1, 22q11.23q12.1(25656238_25925078)x3 | NO | Testicles in scrotal sac, not Müllerian(s) | Atypical genitalia, penoscrotal hypospadias | Male | DSD 46,XY anatomic |
| 1 yr | 46,XY[30] | arr[GRCh37] Xq21.31(88660293_88774857)x2 4q13.2(69435889_69536776)x3, 4q34.3(179062818_179335309)x1, 7q22.1(102101275_102333120)x1, 8p11.22(39247098_39384337)x3, 14q32.33(106272956_106948691)x3 | YES | Testicles in inguinal canal, not Müllerian(s) | Medial hypospadias, Atypical genitalia | Male | DSD 46,XY syndromic |
| 5 yr | 46,XY[30] | arr[GRCh37] 1p13.3(108734222_108853796)x0,1q44(248637251_248790012)x1, 8p11.22(39258176_39386952)x1, 14q32.33(106251487_106706089)x3, 17q12(36283807_36410559)x3, | YES | Unilateral cryptorchidism, not Müllerian(s) | Atypical genitalia | Male | DSD 46,XY syndromic |
| 7 yr | 46,XY[25] | arr[GRCh37] 1p13.3(108734222_108854166)x1,2q12.2q14.1(106212404_117258633)x1-2, 2q13(111366256_113127751)x1, 14q32.33(106329184_106693670)x3, 16q23.3(81982624_82831505)x3, | YES | Bilateral cryptorchidism, Testicles in in inguinal canal, not Müllerian(s) | Proximal Hypospadias | Male | DSD 46,XY syndromic |
| 8 yr | 46,XY[30] | arr[GRCh37] 2q11.2(101022473_101532972)x3, 8p11.22(39250166_39386952)x1,10q26.3(135252898_135377266)x3, 14q32.33(106258365_106709974)x3, 22q11.23q12.1(25656237_25925641)x3, | YES | Testicles in inguinal canal, micropenis | Genital ambiguity | Male | DSD 46,XY syndromic |
| 1.6 yr | 46,XY[20] | arr[GRCh37] 4q13.2(69435889_69541894)x3, 14q11.2(22633668_22940386)x1,14q32.33(106251070_106536858)x3, 16p11.2(29427216_29571495)x1, 16p11.2(32538293_32851881)x1, 22q11.22(23063021_23258369)x3, | NO | Bilateral cryptorchidism, left testicle in inguinal canal, right testicle intra-abdominal | Phenotypic male | Male | DSD 46,XY OT spectrum probably |
| 4 yr | 46,XY[50] | arr[GRCh37] Yq11.23(27488819_27656895)x1, Yp11.2(9927755_10037114)x1, Yp11.2(5505866_5796657)x1, Yp11.2(5222752_5378750)x1, Yp11.2(3893877_4245404)x1, 4q13.2(69367129_69536776)x3,5p15.33(676464_821699)x1, 10p12.33(17935459_18150996)x3, 14q32.33(106207205_106876355)x3, 16p13.11p12.3(16507781_16855348)x3, 17q21.31(44196447_44784639)x3, 22q11.23q12.1(25810488_25922334)x3, | NO | Bilateral Ovotestis in inguinal canal with Mullerian(s) | Atypical genitalia | Male | DSD 46,XY OT |
| 1 yr | 46,XY[20] | arr[GRCh37] 1p13.3(108734222_108853796)x0, 3q26.1(162521297_16262667)x4,8p11.22(39250166_39386952)x1, 10p12.33(17927541_18037328)x1,10q11.22(47587331_48174779)x1, 12p13.31(8004412_8124048)x4, 14q11.2(22746792_22940386)x1, 14q32.33(106272956_106896439)x3,17q12(36283807_36410559)x3,17q21.31(44187492_44784639)x3,22q11.22(23063021_23258369)x3,22q11.21(18916843_19024659)x1 | YES | No gonads | Atypical genitalia, clitoromegaly, hypoplastic labia, closed introit | Female | DSD 46,XY + 22q11.2 deletion syndrome |
| 12 yr | 46,XY[20] | arr[GRCh37] 4q13.2(69435889_69544335)X1,6p25.3(254254_381137)x1,8p11.22(39247098_39384337)x1,13q12.12(25153660_25330022)x3, 14q32.13(94860102_95102132)x3, 14q32.33(106272898_106863508)x3, 16p11.2(32538293_32878155)x1, 16q23.1(76735381_76852275)x1, 17p11.1q11.1(22227470_25274363)x1, 22q11.22(23124375_23258369)x3 | NO | Bilateral cryptorchidism | Atypical Genitalia | Male | DSD 46,XY PAIS probably |
| 9.3 yr | 46,XY[30] | arr[GRCh37] 8p11.22(39247098_39384337)x3,14q32.33(106251070_106536858)x3,16p13.11p12.3(16507781_16855348)x3,22q11.23q12.1(25810488_25922334)x3 | YES | Hydrocolpus in uterus. Testicle in right side | Atypical genitalia | Male | DSD 46,XY OT Spectrum |
| 8 yr | 46,XY[30] | arr[GRCh37]1p13.3(108734223_108853794)x0,2q11.2(97733013_98118115)x1,14q32.33(106329184_106715317)x3,22q11.23q12.1(25656226_25925072)x3, | NO | Right Cryptorchidism left gonad not identifiable | Atypical genitalia | Male | DSD 46,XY OT spectrum |
| 9 m | 46,XY[25] | arr[GRCh37] 1p13.1p11.2(116804531_121339317)x2 6p25.3(294911_380808)x3, 14q32.33(106272956_106536858)x3, 15q26.3(101850435_101947074)x3 | YES | Left cryptorchidism, uterus, anorectal malformation | Atypical genitalia | Not Assigned | DSD 46, XY Syndromic |

DSD: Differences in Sex Development, OT: ovotesticular, PAIS: Partial Androgen Insensitivity Syndrome
